# Bone Marrow Cell Recruitment to the Brain in the Absence of Irradiation or Parabiosis Bias

**DOI:** 10.1371/journal.pone.0058544

**Published:** 2013-03-08

**Authors:** Katrin Kierdorf, Natalie Katzmarski, Carola A. Haas, Marco Prinz

**Affiliations:** 1 Institute of Neuropathology, University of Freiburg, Freiburg, Germany; 2 Faculty of Biology, University of Freiburg, Freiburg, Germany; 3 Department of Neurosurgery, University of Freiburg, Freiburg, Germany; 4 BIOSS Centre for Biological Signalling Studies, University of Freiburg, Freiburg, Germany; University of Lyon, France

## Abstract

The engraftment of bone marrow-derived cells has been described not only during diseases of the central nervous system (CNS) but also under healthy conditions. However, previous studies pointing to an ample bone marrow cell engraftment used irradiation-induced bone marrow chimeras that evoked severe alterations of the CNS micromilieu including disturbances of the blood brain barrier (BBB), damage of endothelial cells and local induction of proinflammatory cytokines. On the other hand, parabiosis experiments using temporarily joined circulatory systems generally yielded low levels of myeloid cell chimerism thereby potentially underestimating bone marrow cell turnover with the CNS. To avoid these drawbacks we established a protocol using the alkylating agent busulfan prior to allogenic bone marrow transplantation from CX_3_CR1^GFP/+^ donors. This regimen resulted in a stable and high peripheral myeloid chimerism, significantly reduced cytokine induction and preserved BBB integrity. Importantly, bone marrow cell recruitment to the CNS was strongly diminished under these conditions and only weakly enhanced during local neurodegeneration induced by facial nerve axotomy. These results underscore the requirement of local CNS conditioning for efficient recruitment of bone marrow cells, establish busulfan as an alternative treatment for studying bone marrow chimeras and suggest a critical re-evaluation of earlier chimeric studies involving irradiation or parabiosis regimens.

## Introduction

Myeloid cells in the central nervous system (CNS) present a highly diverse group of mononuclear cells that shape the local immune response during development and under inflammatory, neoplastic and neurodegenerative conditions [1]–[4]. As such they are critical effector cells and regulators of the innate immune response, the immediate arm of the immune system. The mononuclear phagocytic system of the CNS essentially consists of parenchymal microglia derived from early yolk sac cells as progeny of the early developmental and temporally existing primitive hematopoiesis, whereas other macrophages and monocytes in the circulation are derived from bone marrow stem cells as part of the ontogenetically younger definitive hematopoiesis [5]–[8]. Therefore, microglia with a distinct developmental lineage, compared to other myeloid cells in the CNS, have a negligible exchange with bone marrow or blood cells under normal conditions associated with an intact blood-brain-barrier (BBB) and usually undergo limited self-renewal by local proliferation upon activation [9]. Bone marrow-derived cells on the other hand circulate in the blood as monocytes and populate the CNS as perivascular or meningeal macrophages during steady state conditions and as inflammatory macrophages during autoimmune CNS inflammation [10], [11].

It is still unclear to what extent peripheral myeloid cells engraft in the adult CNS. The answer to this question would have tremendous clinical implications for the treatment of many diseases such as amyotrophic lateral sclerosis (ALS), Alzheimer`s disease (AD), Parkinson`s disease (PD) and many more, since specific myeloid cells could be used as vehicles to deliver neuroprotective or immune relevant genes to the diseased CNS. Priller et al. were among the first who used green fluorescent protein (GFP)-marked bone marrow cells to examine the long-term fate of myeloid cells in the murine CNS after bone marrow transplantation in an experimental setting including whole-body irradiation [12]. Similar to other groups they were able to demonstrate GFP-expressing bone marrow cells in numerous brain regions several weeks after transplantation [13], [14]. With these seminal studies the concept of bone marrow-derived microglia in the CNS was firmly established, in the following years, a plethora of publications appeared which examined the assumed function and fate of bone marrow-derived phagocytes/microglia in different neurological diseases using similar experimental designs. Thus, infiltration of bone marrow-derived myeloid cells was described in disease models with no obvious blood-brain-barrier (BBB) damage such as ALS [15], AD [16], scrapie [17] and many more [18]. Notably, all of these studies used irradiation of the recipient CNS followed by whole bone marrow transplantation. It has been supposed that irradiation might harm the CNS by affecting the integrity of the BBB [19], [20], decreasing tight junction proteins [21] and inducing endothelial cell apoptosis [22]. Indeed, the artificial effect of irradiation on the engraftment of myeloid cells in the CNS was strikingly shown when the heads of recipient mice were protected from irradiation by head shielding [9], [23]. Complementary data on the recruitment of peripheral myeloid cells into the CNS were obtained by a study using parabiosis (in which the circulations of mice are joined) [24]. Despite the fact that this study clearly showed that there were no bone marrow-derived microglia in the CNS of the GFP-negative partner under any tested conditions, the rate of chimerism in the parabiosis model is generally much lower compared to irradiation which might lead to an underestimation of myeloid cell engraftment.

Here we established an alternative model of bone marrow chimeras which does not require irradiation or parabiosis. We demonstrate that bone marrow cell ablation via the alkylating agent busulfan leads to an unexpected high and stable grade of blood chimerism with strongly reduced inflammatory responses in the CNS compared to irradiation regimens. Moreover, BBB alterations after busulfan treatment were only minimal. Under these conditions myeloid cell recruitment under non-diseased conditions was merely subtle and only minimally enhanced during neurodegeneration. Thus, our data highlight the role of host endogenous factors for bone marrow cell entry into the CNS and provide a new model for studying myeloid cell turnover during neurodegeneration.

## Results

### Reduced Inflammatory Response in the Brain and BBB Leakage after Busulfan Treatment

Several pharmacological strategies are currently being used for patient treatment before bone marrow transfer. For example, the bifunctional alkylating agent busulfan is applied in combination with cyclophoshamide as conditioning regimen prior to allogenic hematopoietic progenitor cell transplantion for chronic myelogenous leukemia [25].

**Figure 1:**
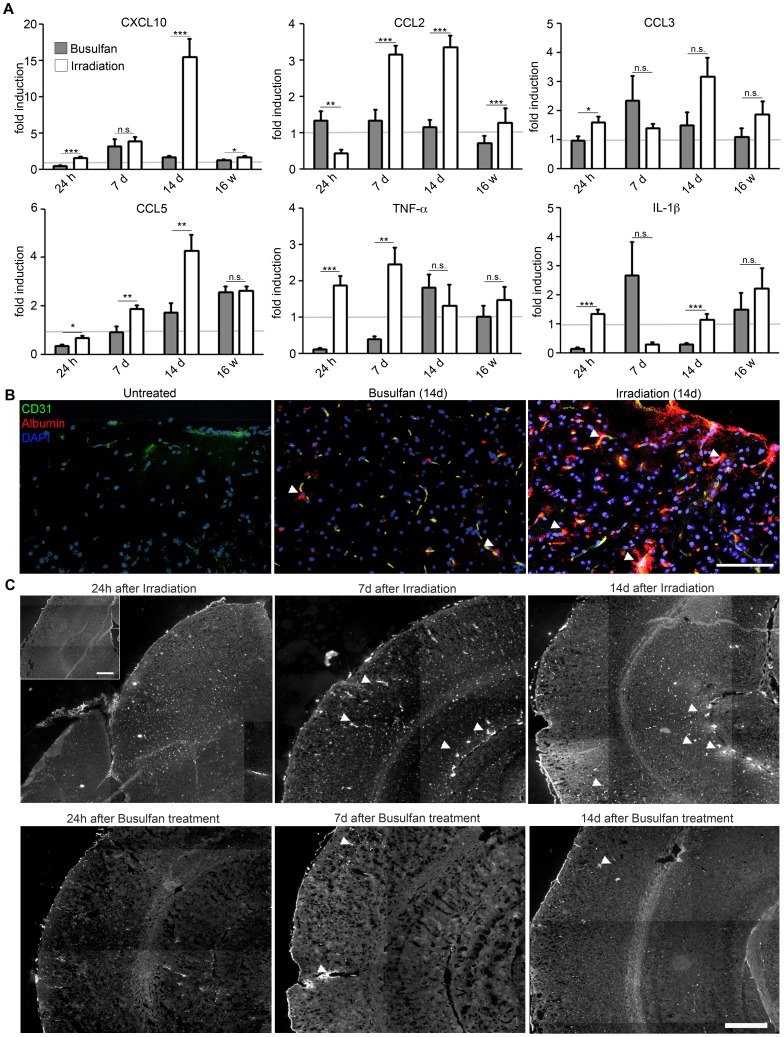
Reduced conditioning of the CNS after busulfan treatment. **A)** Strongly diminished induction of proinflammatory cytokines and chemokines in busulfan-treated chimeras compared to irradiation protocols. Quantitative real-time PCR analysis of cytokine and chemokine induction in brains of busulfan-treated (grey columns) and whole-body irradiated (white columns) chimeras 24 hours (h), 7 days (d), 14 d and 16 weeks (w) after treatment. The mRNA expression was normalized to GAPDH and compared to untreated mice, indicated by the grey line. Data are shown as mean ± SEM. One out of two experiments is shown with three to six animals per group. Statistical significance is marked with asterisks (p<0.05 = *; p<0.01 = **; p<0.001 = ***). **B)** Largely preserved blood-brain-barrier (BBB) integrity after busulfan challenge. Direct fluorescence microscopic visualization depicting CD31^+^ endothelial cells (green), extravasal albumin (red) and nuclei (DAPI, blue). Arrow heads point to extravasated albumin. Representative pictures of cortices are shown. Scale bar = 100 µm. **C)** Albumin staining of the CNS parenchym is shown for both treated groups 24 hours (h), 7 days (d) and 14 d after treatment. Arrow heads point to extravasated albumin in superficial and deeper brain regions. Representative pictures are shown. Scale bar = 400 µm. Insert: Albumin staining of an untreated animal. Scale bar = 400 µm.

To test whether busulfan can also be used for bone marrow cell ablation in rodents we challenged mice with 30 µg/g body weight busulfan prior to bone marrow cell transfer. It has been reported that irradiation as an alternative method induced a tremendous and rapid gene induction in the CNS [9], [23]. We therefore compared the time-dependent induction of several myeloattractants and of the proinflammatory cytokines TNFα and IL-1β in the CNS (hippocampus, cortex and thalamic area) of irradiated and busulfan-treated mice (**Fig. 1A**). Notably, we observed a strong induction of cytokines and chemokines after treatment in irradiated brains but much less in busulfan-challenged animals (CXCL10 after 14 days: 15.44±2.48 fold increase after irradiation versus 1.67±0.20 fold increase after busulfan, *P*<0.001; CCL2 after 14 days: 3.35±0.32 fold increase after irradiation and 1.16±0.20 fold increase after busulfan, *P*<0.001; CCL5 after 14 days: 4.29±0.65 fold increase after irradiation versus 1.72±0.39 fold increase after busulfan, *P* = 0.004; IL-1β after 14 days: 1.15±0,19 fold increase after irradiation versus 0.30±0.03 fold increase after busulfan, *P*<0.001). The significant induction of CCL2, the major chemoattractant for myeloid cells [26], coincides with the described appearance of GFP-expressing cells in perivascular and leptomeningeal sites 7–14 days after bone marrow transplantation [12]. Interestingly, the proinflammatory cytokines IL-1β and TNFα that have multiple effects on endothelial cell function [27] were rapidly induced after 24 h in the irradiated group (IL-1β after 24 h: 1.34±0.15 fold increase, *P*<0.001; TNFα after 24 h: 1.88±0.26 fold increase, *P* = 0.001) and reduced after busulfan exposure (IL-1β after 24 h: 0.14±0.05 fold increase, *P*<0.001; TNFα after 24 h: 0.12±0.04 fold increase, *P*<0.001).

To examine the functional consequences of the treatment-induced elevated cytokine and chemokine levels, we next evaluated the integrity of the BBB. Extravascularly localized albumin as morphological correlate for BBB leakage was therefore visualized by means of immunohistochemistry 24 h, 7 d and 14 d after treatment (**Fig. 1B and 1C**). No apparent disruption of the BBB was observed in busulfan-treated brains at all time points analysed whereas massive albumin extravasations were clearly detectable in irradiated brains 7 d and 14 days after challenge. However, 24 h after irradiation we could not detect any obvious albumin leakage into the CNS parenchym. In sum, bone marrow ablation in mice by busulfan does not induce a robust production of inflammatory mediators that lead to overt changes in brain vasculature.

### Successful Delivery of GFP^+^ Hematopoietic Cells by Autologous Bone Marrow Reconstitution after Busulfan Pre-treatment

**Figure 2:**
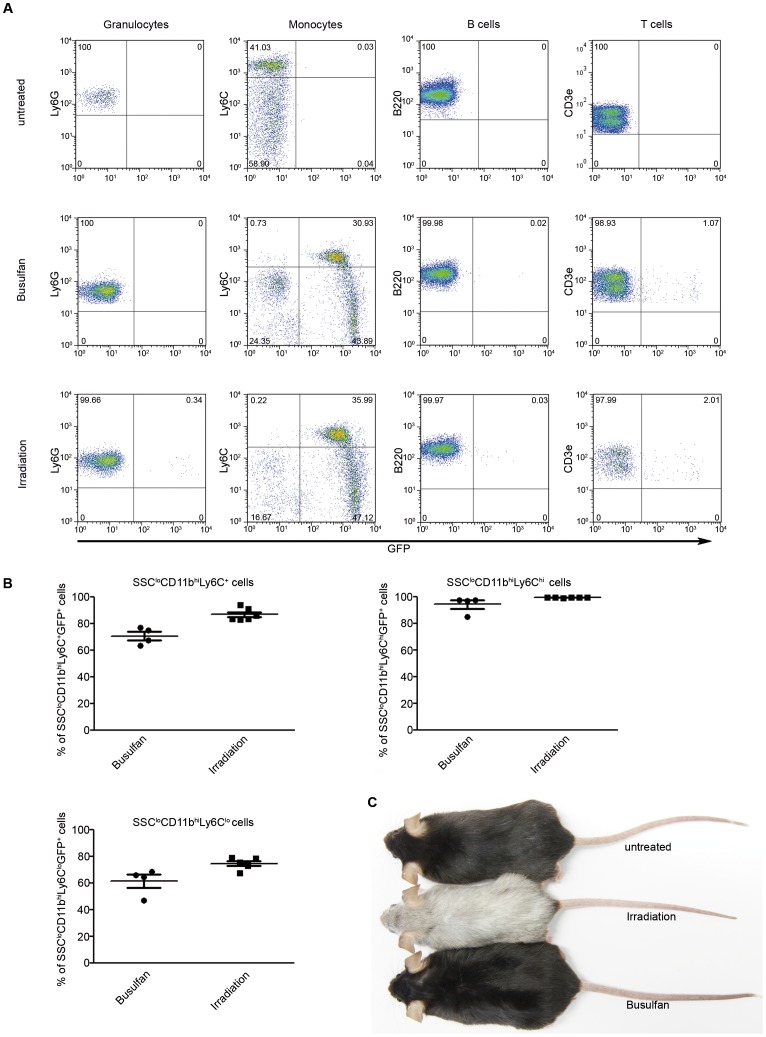
High peripheral blood myeloid chimerisms in busulfan-treated chimeras. **A)** Representative FACS dot plots showing the expression of GFP in blood granulocytes, monocytes, B cells and T cells of busulfan-treated and irradiated CX_3_CR1^GFP/+^ → CX_3_CR1^+/+^ chimeras and untreated mice four weeks after bone marrow transfer. Percentages of the respective cell populations are indicated. CX_3_CR1^+/+^ mice served as untreated controls. **B)** Quantitative assessment of GFP^+^ expression in overall monocytes (SSC^lo^CD11b^+^) and the two monocyte subsets SSC^lo^CD11b^+^Ly6C^hi^ and SSC^lo^CD11b^+^Ly6C^lo^ out of all SSC^lo^CD11b^+^ cells. Data depict high reconstitution efficiencies and comparable chimerisms in both groups. One symbol represents one mouse (Busulfan-treated: filled circle; irradiated: filled squares). Mean ± SEM are shown. Six to seven animals per group were analysed. **C)** Photograph showing change of fur color 16 weeks after treatment. Irradiation leads to a loss of fur color, whereas busulfan-treated animals remain unaffected. Top: untreated animal, middle: whole-body irradiated animal, bottom: busulfan-treated mouse.

In order to distinguish invading bone marrow-derived phagocytes from brain endogenous myeloid cells, bone marrow chimeras were created as described previously [9], [23]. As donors we utilized bone marrow from CX_3_CR1^GFP/+^ transgenic mice which express enhanced green fluorescent protein (GFP) in the circulation especially in myeloid cells, but not in granulocytes. Four weeks after transplantation GFP-marked peripheral blood cells were analyzed by FACS analysis. As expected, the rate of GFP-marked cells in CX_3_CR1^GFP/+^ → CX_3_CR1^+/+^ chimeras was high in irradiated mice for Ly6C^hi^ (approximately 36%) and Ly6C^lo^ monocytes (47%) and much lower or not present in granulocytes (Ly6G), B cells (B220) and T cells (CD3e) mimicking endogenous gene expression (CD3, **Fig. 2A**). Surprisingly, busulfan treatment resulted in an almost equally high rate of chimerism with approximately 31% GFP-labelled Ly6C^hi^ and 44% GFP^+^ Ly6C^lo^ monocytes. Detailed evaluation of GFP expression in the myeloid compartment revealed high chimerism in all monocytes (SSC^lo^CD11b^+^) after busulfan treatment in general (70.78±3.12% compared to 86.89±1.86% upon irradiation) and especially in SSC^lo^CD11b^+^Ly6C^hi^ “inflammatory” monocytes (94.39±3.18% compared to 99.47±0.06% upon irradiation, **Fig. 2B**). The chimerism in SSC^lo^CD11b^+^Ly6C^lo^ “patrolling” monocytes was reduced under the non-irradiation regimen (61.51±4.88% compared to 74.61±1.74% upon irradiation). We had previously described the loss of mouse fur color as an obvious macroscopical sign of irradiation-induced skin damage [9] most likely due to damage of dermal melanocytes [28]. Importantly, the alkylating agent busulfan did not induce any changes of fur color compared to irradiation protocols suggesting less dermal side effects (**Fig. 2C**). Of note, GFP expression in the busulfan group was stable over the entire observation period of up to five months, suggesting that bone marrow reconstitution led to stable hematopoietic engraftment.

### Drastically Reduced Bone Marrow Cell Recruitment to the CNS Under Non-diseased Conditions

**Figure 3:**
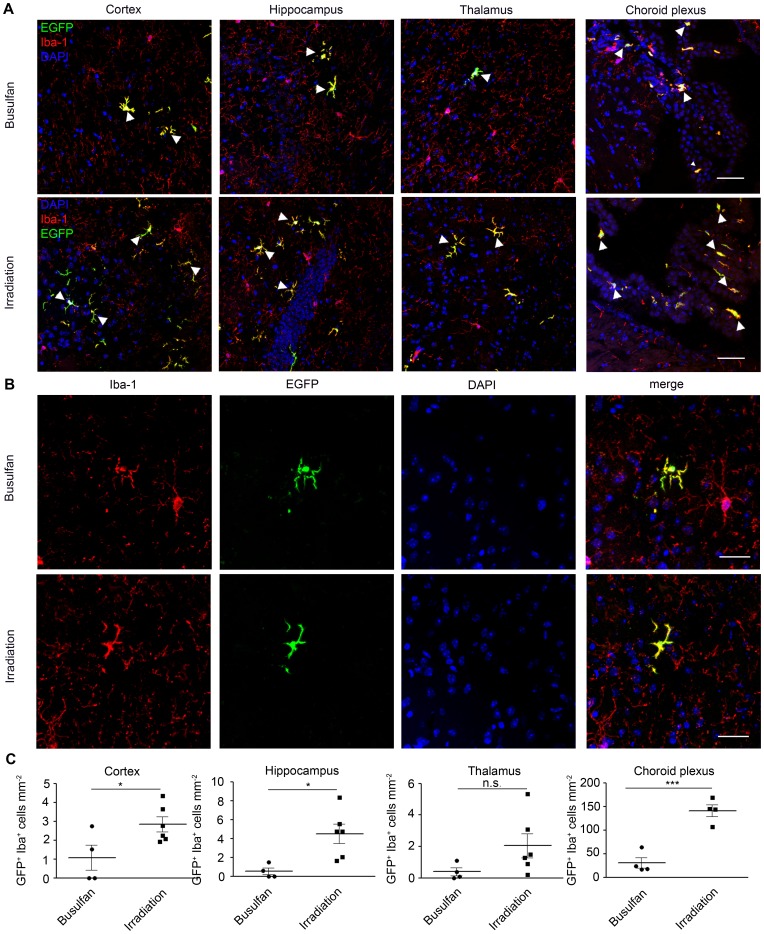
Engraftment of donor-derived GFP^+^ bone marrow cells into the brain strongly reduced in busulfan-treated animals. The number of engrafted GFP^+^Iba-1^+^ donor-derived phagocytes is strongly decreased in the busulfan-treated animals compared to irradiated mice. **A)** Immunohistochemistry of chimeric brains shows GFP^+^Iba-1^+^ cells in the cortex, hippocampus, thalamus and choroid plexus of busulfan-treated and irradiated chimeric mice 16 weeks after reconstitution. Arrow heads indicate representative ramified GFP^+^ (green) and Iba-1^+^ (red) cells of donor origin in the CNS. Nuclei are counterstained with DAPI (blue). Bars = 200 µm. **B)** High magnification images of donor-derived GFP^+^ Iba1^+^ reveal ramified morphology of engrafted cells in both groups. Sections are stained for Iba-1 (red), CX_3_CR1 (GFP) and DAPI (blue). Scale bars = 50 µm. **C)** Quantification of ramified GFP^+^Iba-1^+^ cells in cortex, hippocampus, thalamus and choroid plexus. Each symbol represents one mouse. Data show significant differences in the number of engrafted GFP^+^Iba-1^+^ donor-derived cells in all investigated brain areas between the busulfan-treated (filled circle) and irradiated (filled squares) animals 16 weeks after bone marrow cell transfer. Asterisks indicate statistical significance (p<0.05 = *; p<0.01 = **; p<0.001 = ***). All graphs show mean ± SEM.

After having established a new and robust protocol for labelling donor BM cells without artificial irradiation steps, we histologically examined brain sections from chimeric mice several weeks after bone marrow transplantation (**Fig. 3**).

As expected, we could easily find some elongated GFP^+^ cells in the choroid plexus of chimeric mice which had received irradiation treatment (**Fig. 3** **A,B**). These cells were closely vessel-associated, resembling blood-derived choroid plexus macrophages and perivascular cells. In mice which received busulfan treatment prior to bone marrow cell transfer, on the other hand, the amount of donor-derived fusiform GFP-expressing cells was dramatically decreased (30.44±11.23 cells/mm^2^ and 140.9±12.73 cells/mm^2^ after irradiation, *P = 0.006*). Our main focus, however, were parenchymal cells expressing the marker GFP. Microscopical investigation of the cortex, hippocampus and thalamus 16 weeks after transplantation revealed some parenchymal cells with typical microglial morphology such as round to spindle-shaped soma and a distinct arborization pattern. These cells were positive for the microglia/macrophage marker Iba-1. Notably, significant bone marrow cell engraftment occurred only in regions of the brains that were conditioned by irradiation (**Fig. 3C**). Only few, if any, parenchymal GFP^+^Iba-1^+^ cells were detectable in busulfan-treated brains (cortex: 1.07±0.66 cells/mm^2^ after busulfan and 2.83±0.39 cells/mm^2^ after irradiation, *P = *0.04; hippocampus: 0.51±0.34 cells/mm^2^ after busulfan and 4.50±1.01 cells/mm^2^ after irradiation, *P = *0.015; thalamus: 0.39±0.25 cells/mm^2^ in busulfan versus 2.05±0.77 cells/mm^2^, *ns* in irradiated mice). Few GFP^+^ Iba-1^+^ perivascular macrophages were rarely detectable in the cortex of both irradiated and busulfan-treated animals (**Fig. S1**). These data reinforce our previous reports of irradiation induced migration of bone marrow cells [9], [23] and provides evidence that the alternative approach of using busulfan leads to drastically reduced numbers of engrafted cells despite high and stable blood chimerism.

### No Frank Myeloid Cell Engraftment during CNS Pathology in Busulfan Chimeras

To investigate whether the generation of GFP^+^ phagocytes/microglia from CX_3_CR1^GFP/+^ transgenic bone marrow is enhanced during CNS pathology and whether cell recruitment into the diseased brain requires an irradiation-induced rather than a busulfan-primed host environment, we induced neurodegeneration in adult mice using the facial nerve axotomy model. In this experimental procedure the BBB remains intact and a remote degeneration of neurons in the facial nucleus leads to local microglia activation with concomitant recruitment of bone marrow cells in whole-body irradiated mice [12].

**Figure 4:**
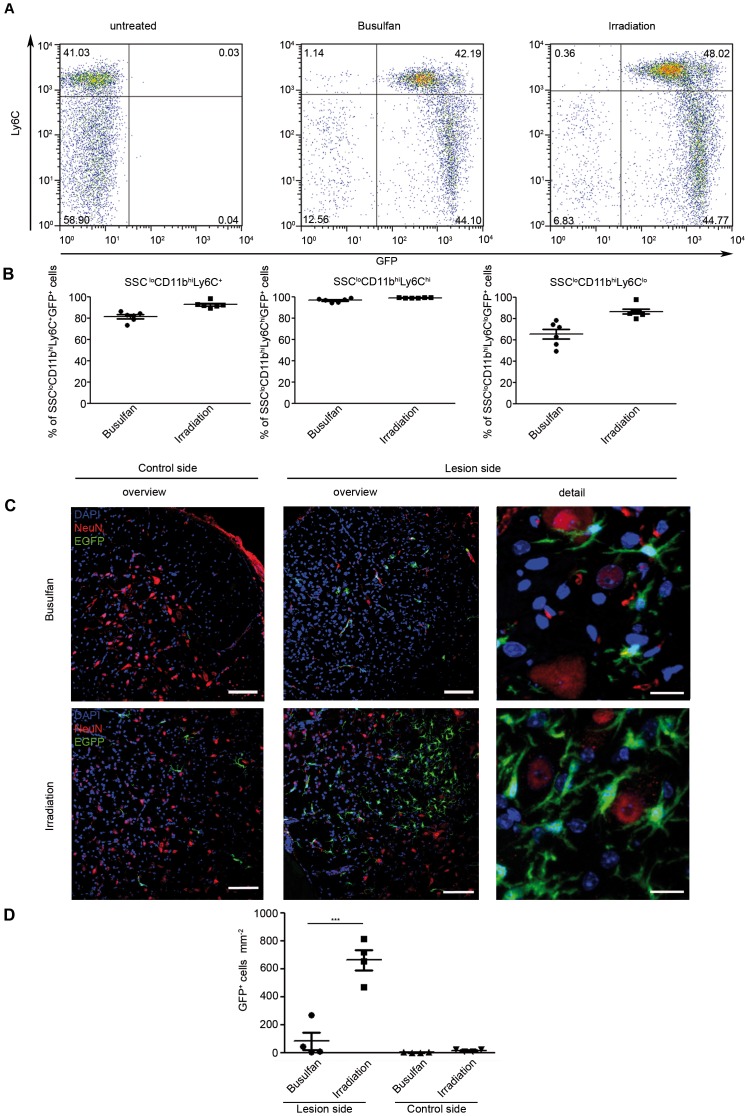
Recruitment of donor-derived GFP^+^ bone marrow cells into the lesioned brain depends on irradiation. Following facial nerve axotomy, the recruitment of GFP^+^Iba-1^+^ donor-derived bone marrow cells to the lesioned N. facialis is strongly diminished in busulfan-treated mice compared to irradiated animals. **A)** Representative FACS dot plots of peripheral blood in the SSC^lo^CD11b^+^ monocyte compartment in untreated, busulfan-treated and irradiated mice. **B)** Quantification of the peripheral blood chimerism shows sufficient and comparable reconstitution levels in busulfan-treated (filled circles) and irradiated mice (filled squares). One symbol represents one mouse. Chimerism was analyzed nine weeks after reconstitution. Six animals per group were analysed. All graphs show mean ± SEM. **C)** Immunohistochemistry of recruited GFP^+^Iba-1^+^ phagocytes in the lesioned facial nucleus two weeks following axotomy. Elevated numbers of ramified donor-derived GFP^+^ cells were found in the degenerating neurons (NeuN, red) of irradiated CX_3_CR1^GFP/+^ → CX_3_CR1^+/+^ mice compared to busulfan-treated CX_3_CR1^GFP/+^ → CX_3_CR1^+/+^ animals. GFP^+^ cells were in close proximity to NeuN-immunoreactive neurons in the facial nucleus. No GFP expressing cells were found on the control site in busulfan-treated mice, in contrast to irradiated mice where a few GFP^+^ cells could be found. Scale bars: overview = 200 µm; detail = 50 µm. Nuclei were stained with DAPI (blue). **D)** Quantification of engrafted GFP^+^ Iba-1^+^ cells 14 days after axotomy. A robust engraftment of donor-derived GFP^+^ cells was found in irradiated chimeric mice (filled squares), whereas only few GFP^+^ cells were detectable in busulfan-treated chimeric mice (filled circle). Symbols indicate individual mice. Data are expressed as mean ± SEM. Asterisks indicate statistical differences (p<0.001 = ***).

Importantly, only chimeric busulfan-treated and irradiated CX_3_CR1^GFP/+^ → CX_3_CR1^+/+^ chimeras with a high and stable degree of chimerism were used for facial nerve axotomy (**Fig. 4A,B**). During the experiments ramified GFP-expressing cells were found in close proximity to the lesioned facial motoneurons 14 days after axotomy (**Fig. 4C,D**). In contrast, the unlesioned contralateral facial nucleus was largely devoid of engrafted GFP^+^ cells. Again, high recruitment of donor-derived bone marrow cells to the side of neurodegeneration took place when the brain was conditioned by irradiation. In contrast, busulfan-treated brains contained much less recruited cells. Upon quantification we found significantly more ramified GFP^+^Iba-1^+^ cells in the lesioned facial nucleus in irradiated mice (663.1±72.38 cells/mm^2^) than in busulfan-treated animals (81.66±63.31 cells/mm^2^, *P = *0.001). Interestingly, few engrafted cells were also found in the contralateral facial nucleus in irradiated but not busulfan-treated mice (15.07±4.84 cells/mm^2^ after irradiation and 1.56±0.98 cells/mm^2^, *P = *0.037 in busulfan-treated animals). These results underscore that brain damage is required for sufficient bone marrow cell engraftment in the healthy and even in diseased brains with intact blood-brain-barriers.

## Discussion

In this study we established the usage of busulfan as an appropriate and less toxic option for preparing bone marrow chimeras in mice. Application of busulfan rather than irradiation led to a highly efficient and stable peripheral chimerism that allowed for studying myeloid cell turnover into the lesioned and healthy CNS. We found overwhelming evidence that busulfan, in contrast to irradiation, leads to only mild artificial changes of the CNS microenviroment with highly preserved BBB integrity and only subtle local inflammatory responses.

Despite the fact that busulfan was observed to be less harmful for the CNS compared to irradiation in our study, it is potentially toxic. Busulfan is a functional alkylating agent in which two labile methanesulonate groups are attached to opposite ends of a four-carbon alkyl chain. In aqueous media, busulfan hydrolyzes to release the methansulfonate groups. producing reactive carbonium ions that can alkylate DNA [29]. This DNA damage is thought to be responsible for much of the cytotoxicity of busulfan such as mutagenic and clastogenic effects [30]. However, in our experiments we did not observe any lethal effects, changes of fur color our induction of tumors in the busulfan-treated animals up to the end of the observation period, e.g. 12 months of age. In fact, the chemotherapy protocol we applied was the same that had already been used to successfully treat patients in a gene therapy study of X-linked adrenoleukodystrophy [31].

We report that a lethal dose of whole-body irradiation has dramatic effects on the brain micromilieu as evidenced by the strong induction of inflammatory mediators and obvious BBB changes. These observations are in line with previous reports on harmful effects of irradiation on the CNS [9], [23]. However, in contrast to a previous study [32], we found that irradiation induces massive changes of the BBB 7 d and 14 d after treatment. The authors analysed irradiation-induced changes in the CNS already 6 h and 48 h after irradiation and did not find any albumin or IgG leakage into the parenchyma. In fact, we also did not observe any apparent BBB disruption 24 h after treatment that suggests that any functional alterations may occur at later time points. We could also show in a previous study that this dosage is already sufficient to induce strong and even long-lasting changes of microglia morphology and cytokine profile thereby strongly changing brain homeostasis [23]. It was surprising to find that these alterations persist for several months after irradiation, indicating major effects of this conditioning regimen. Finally, numerous reports in several species have already described detrimental effects of irradiation on vascular endothelial cells thus potentially allowing myeloid cell recruitment into the brain [21], [33].

Taken together, our results indicate that brain-entry permitting irradiation induces long-lasting side effects inside the brain which precludes, at least in our opinion, the use of brain irradiation in a clinical setting. Whether brain-related side effects can be seen in other clinical settings, such as for the treatment of different types of leukemia, remains to be proven.

Our results with busulfan confirm those observed in the models of irradiated mice with a protected head [9], [23] in that it appears that irradiation has a modulating effect on the CNS that allows for the entry of bone marrow-derived myeloid cells into the tissue. Plenty of grafted cells were found in brain regions devoid of the BBB such as the choroid plexus. The choroid plexus has been shown to be an important cellular entry site for immigrating encephalitogenic cells during CNS autoimmunity thereby acting as a door keeper enabling the immune privilege of the brain [34].

What is the relevance of our findings for patients suffering from neurodegeneration? There are several reports indicating that approximately 60% of patients with AD have a disturbed BBB [35], [36]. Moreover, AD patients are generally aged and often have a history of cerebrovascular events caused by cerebral amyloid angiopathy, including ischemic insults [37]. Therefore, it can be assumed that circulating mononuclear phagocytes may engraft in the brains in AD patients without additional conditioning by irradiation. In this setting, we believe that bone marrow transfer with protocols including busulfan might be suitable to achieve high chimerism and to get access to the diseases brain with only minimal additional damage. Thus, this might open new treatment options for the short or long-term treatment of this group of CNS diseases.

## Materials and Methods

### Mice and Generation of Bone Marrow Chimeric Mice

Bone marrow chimeric mice were generated as described recently [23]. Eight week old C57Bl/6J recipient mice were reconstituted with 5×10^6^ bone marrow cells derived from femur and tibia of adult CX_3_CR1^GFP/+^ mice and injected into the tail vein of recipients. Recipients were conditioned either by whole-body irradiation or treatment with the chemotherapeutic agent busulfan. Mice received whole-body irradiation (11Gy) 24 h prior to bone marrow reconstitution with an RS 2000 Biologica x-Ray irradiator. Mice treated with busulfan received three intraperitoneal injections of 30 µg/g body weight 7, 5 and 3 days prior to bone marrow transfer. All mice were treated with antibiotics (Trimethoprim and Sulfamethoxazol (Cotrim-K®) for 14 days after irradiation or busulfan treatment.

Ethics statement: The animal experiments were performed in accordance with the guidelines of Bezirksregierung Freiburg legislation for animal experiments. The respective animal permission number is G-87.007.

Four and nine weeks after bone marrow reconstitution, peripheral blood chimerism was assessed by FACS analysis. Blood samples were prepared in buffer solution (PBS containing 2% FCS and 2% EDTA) at 4°C and stained with anti-B220 (Becton Dickinson), anti-CD11b, anti-CD3ε, anti-Ly-6C and anti-Ly-6G (all eBioscience). After red blood cell lysis (RBC lysis buffer, eBioscience) single cell suspensions were analyzed on a FACS Canto II (Becton Dickinson). Data were acquired with FlowJo.

### Quantification of Microglia/Macrophage Engraftment in the CNS

The CNS of chimeric mice was analyzed 12 and 16 weeks after bone marrow reconstitution. After transcardial perfusion with phosphate-buffered saline (PBS), brains were fixed in 4% paraformaldehyde, 14 µm cryosections were obtained as described previously [18]. Sections were then blocked with PBS containing 5% bovine serum albumin. Primary antibodies were added over night at a dilution of 1∶800 for albumin, 1∶300 for CD31, 1∶300 for Iba-1, 1∶500 for NeuN. Sections stained for Iba-1 or NeuN were permeabilized with 0.1% Triton-X 100 in PBS. Secondary antibodies were added as follows: Alexa Flour® 488 1∶800 and Alexa Flour® 555 1∶800 for 90 min at RT. Nuclei were counterstained with DAPI. GFP-expressing and Iba-1^+^ macrophages/microglia were counted in at least three sections of each individual animal according to earlier protocols [9], [23]. The number of cells and the examined area were determined microscopically using a conventional fluorescence microscope (Olympus BX-61) and the confocal pictures were taken with Fluoview FV 1000 (Olympus).

### Facial Nerve Axotomy

Unilateral facial nerve axotomy was induced in bone marrow chimeric mice 9 weeks post-transplantation by transection of the facial nerve at the stylomastoid foramen, resulting in ipsilateral whisker paresis as described previously [9], [12]. Animals were killed for analysis 2 weeks after transection.

### Real-time PCR

RNA was extracted from brains (hippocampus, cortex and thalamic area) at indicated time points after busulfan injection or irradiation, respectively. RNA was isolated using RNAeasy Mini kit (Quiagen, Hilden, Germany) following the manufacturer’s instructions as described previously [38]. The samples were then treated with DNAseI and 1 µg of RNA was transcribed into cDNA using oligo-dT primers and the SuperScript II RT kit (Invitrogen, Carlsbad, CA). 2.5 µl cDNA was subsequently transferred into a 96-well Multiply® PCR-plate (Sarstedt, Germany) and 12,5 µl SYBR green® master Mix plus 9.6 µl H_2_O were added. The house keeping gene GAPDH was used as reference gene. The following primer pairs were used: CCL2 5′-TCT GGG CCT GCT GTT CAC C-3′ and 5′-TTG GGA TCA TCT TGC TGG TG-3′; CCL3 5′-CAC CAC TGC CCT TGC TGT T-3′ and 5′-AGG AGA AGC AGC AGG CAG TC-3′; CCL5 5′-TGC CCA CGT CAA GGA GTA TTT-3′ and 5′-TCT CTG GGT TGG CAC ACA CTT-3′; CXCL10 5′-TGC TGG GTC TGA GTG GGA CT-3′ and 5′-CCC TAT GGC CCT CAT TCT CAC-3′; TNF-α 5′-CAT CTT CTC AAA ATT CGA GTG ACA A-3′ and 5′-TGG GAG TAG ACA AGG TAC AAC CC-3′; IL-1β 5′-ACA AGA GCT TCA GGC AGG CAG TA-3′ and 5′- ATA TGG GTC CGA CAG CAC GAG-3′; GAPDH 5′-TCC TGC ACC ACC AAC TGC TTA GCC-3′ and 5′-GTT CAG CTC TGG GAT GAC CTT GCC-3′.

### Statistical Analysis

Results were analysed with Prism 4.0 (GraphPad) and statistical differences were evaluated using a non-paired Student`s *t* test. Significant differences were marked by using asterisks (p<0.05 = *; p<0.01 = **; p<0.001 = ***). All graphs show means ± SEM.

## Supporting Information

Figure S1 **GFP^+^ Iba-1^+^ perivascular macrophages (PVMs) are rarely detected in irradiated (upper panels) and busulfan treated animals (lower panels).** Double labeled PVMs for GFP (green) and Iba-1 (red) are indicated by asterisks. Nuclei counterstaining with DAPI is shown in blue. Scale bar: 50 µm.
(TIF)
