# Supplementary figures and images for: Bone Marrow Cell Recruitment to the Brain in the Absence of Irradiation or Parabiosis Bias
Source: PLoS One. 2013 Mar 8;8(3):e58544. doi: 10.1371/journal.pone.0058544 (PMC3592806; doi:10.1371/journal.pone.0058544)

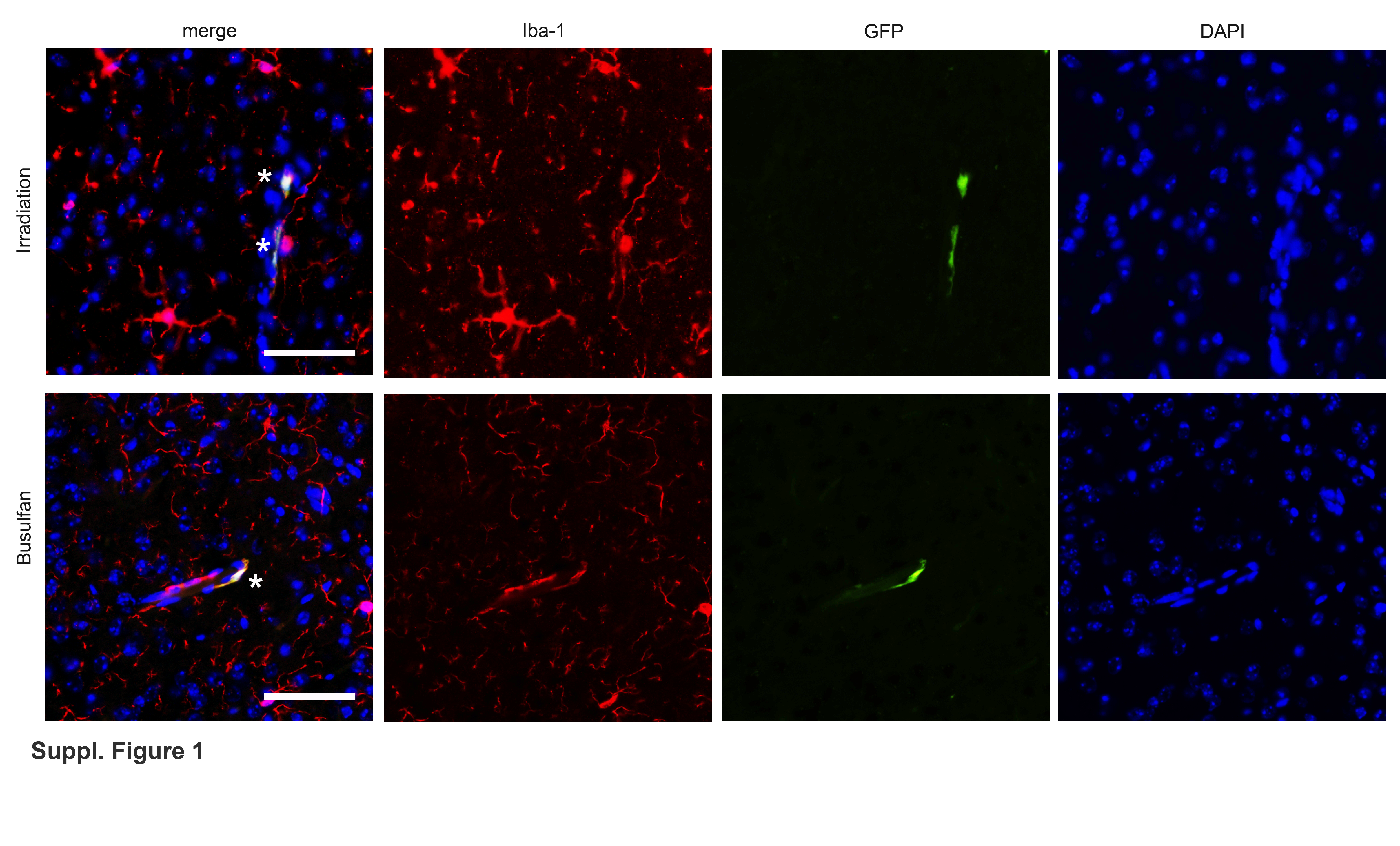

Supplement: Figure S1 — GFP+ Iba-1+ perivascular macrophages (PVMs) are rarely detected in irradiated (upper panels) and busulfan treated animals (lower panels). Double labeled PVMs for GFP (green) and Iba-1 (red) are indicated by asterisks. Nuclei counterstaining with DAPI is shown in blue. Scale bar: 50 µm. (TIF) [file pone.0058544.s001.tif]
